# Flexible Force Sensor Based on a PVA/AgNWs Nanocomposite and Cellulose Acetate

**DOI:** 10.3390/s24092819

**Published:** 2024-04-28

**Authors:** Dulce Natalia Castillo-López, Luz del Carmen Gómez-Pavón, Alfredo Gutíerrez-Nava, Placido Zaca-Morán, Cesar Augusto Arriaga-Arriaga, Jesús Manuel Muñoz-Pacheco, Arnulfo Luis-Ramos

**Affiliations:** 1 Grupo de Sistemas Fotónicos y Nanoóptica, Facultad de Ciencias de la Electrónica, Benemérita Universidad Autónoma de Puebla, Puebla 72570, Mexico; dulce.castillolo@correo.buap.mx (D.N.C.-L.); alfredo.gutierrezn@alumno.buap.mx (A.G.-N.); cesarau.arriaga@correo.buap.mx (C.A.A.-A.); jesusm.pacheco@correo.buap.mx (J.M.M.-P.); arnulfo.luis@correo.buap.mx (A.L.-R.); 2 Instituto de Ciencias, Ecocampus Valsequillo, Benemérita Universidad Autónoma de Puebla, Puebla 72960, Mexico; placido.zaca@correo.buap.mx

**Keywords:** force sensor, PVA, silver nanowires, cellulose acetate, flexible

## Abstract

Nanocomposites are materials of special interest for the development of flexible electronic, optical, and mechanical devices in applications such as transparent conductive electrodes and flexible electronic sensors. These materials take advantage of the electrical, chemical, and mechanical properties of a polymeric matrix, especially in force sensors, as well as the properties of a conductive filler such as silver nanowires (AgNWs). In this work, the fabrication of a force sensor using AgNWs synthesized via the polyol chemical technique is presented. The nanowires were deposited via drop-casting in polyvinyl alcohol (PVA) to form the active (electrode) and resistive (nanocomposite) sensor films, with both films separated by a cellulose acetate substrate. The dimensions of the resulting sensor are 35 mm × 40 mm × 0.1 mm. The sensor shows an applied force ranging from 0 to 3.92 N, with a sensitivity of 0.039 N. The sensor stand-off resistance, exceeding 50 MΩ, indicates a good ability to detect changes in applied force without an external force. Additionally, studies revealed a response time of 10 ms, stabilization of 9 s, and a degree of hysteresis of 1.9%. The voltage response of the sensor under flexion at an angle of 85° was measured, demonstrating its functionality over a prolonged period. The fabricated sensor can be used in applications that require measuring pressure on irregular surfaces or systems with limited space, such as for estimating movement in robot joints.

## 1. Introduction

Currently, research in the field of flexible electronics is focused on applications for force measurement systems using nanomaterials. The flexibility and ability of these devices to withstand mechanical deformations make them a viable alternative compared to conventional electronic components, which are typically rigid. This technological advancement has found applications in various sectors, such as the automotive industry [1], biomedical field [2,3], and the development of robotic skins [3,4,5], among others.

Recent studies on these types of systems have shown notable characteristics, such as high sensitivity [3,6], stretchability [6], response rate [3,6], stability [6], and low fabrication cost [6]. Regarding force sensors [7,8,9], they can employ various measurement techniques based on phenomena such as piezoelectric, piezocapacitive, triboelectric, or piezoresistive [1,6]. In particular, piezoresistive pressure sensors transform input pressure into a change in resistance in the device [6]. These sensors represent one of the most investigated sensing systems due to their low power consumption and high sensitivity in the low-pressure range [6,10,11].

Expectations regarding future technologies currently focus on the design of devices based on nanomaterials and polymers, with silver nanowires (AgNWs) being a prominent nanomaterial due to their thermal [12,13], optical [12,14], and electrical properties [12,13], as well as their application in transparent electrodes [15,16], optical polarizers [17,18], biomolecular sensors [3,19], catalysis, and batteries [20]. Taking advantage of the mechanical properties of the nanowires, piezoresistive sensors (PRSs) have been developed [21,22,23], as they exhibit good stability [24], durability, and flexibility, which are characteristics that conventional PRSs lack [24]. This has driven the development of flexible PRSs and electronic systems composed of nanocomposites that combine an elastic matrix (polymeric) and metallic nanomaterials [7,23]. Here, polymers play an important role as a polymeric matrix in these nanocomposites, providing the device with portability, stretchability, stability, and flexibility without compromising the flexibility and performance of the metallic filler [3,25]. Flexible PRSs focus on flexibility, low detection limits, low cost, and versatility, making them the most popular choice for applications in flexible electronics, such as portable health monitors, electronic skins, and biomedical diagnostics [3,23]. 

This work reports the fabrication of a flexible piezoresistive sensor using AgNWs in a polyvinyl alcohol (PVA) matrix. The active and resistive films are deposited on PVA via drop-casting and separated by a cellulose acetate substrate, ensuring an electrical response proportional to the applied force. The novelty of the sensor lies in the utilization of a composite comprising PVA and AgNWs, leveraging the mechanical stability of PVA and the electrical conductivity of AgNWs. As a result, the operating range obtained is from 0.039 to 3.92 N, with a stable high sensitivity and a response time of 10 ms. This design combines flexibility and adaptability to provide accurate measurements of force and pressure on irregular surfaces or in confined spaces. Particularly, this sensor holds potential applications in biomechanical and portable sensor technology, representing a significant contribution to the field of piezoresistive sensors.

## 2. Materials and Methods

The development of the flexible force sensor started with the synthesis of the AgNWs, which were made by using the polyol technique [26,27]. These nanowires were subsequently used to fabricate the resistive film and the electrode. Initially, the AgNWs were deposited over polyethylene terephthalate (PET) substrates to later transfer them to a polymeric matrix and thus obtain the resistive films. Simultaneously, the electrode was designed in a predetermined pattern. Finally, both manufactured flexible polymeric nanocomposite films fabricated were joined, placing a separator between them.

### 2.1. Synthesis of AgNWs

**Figure 1:**
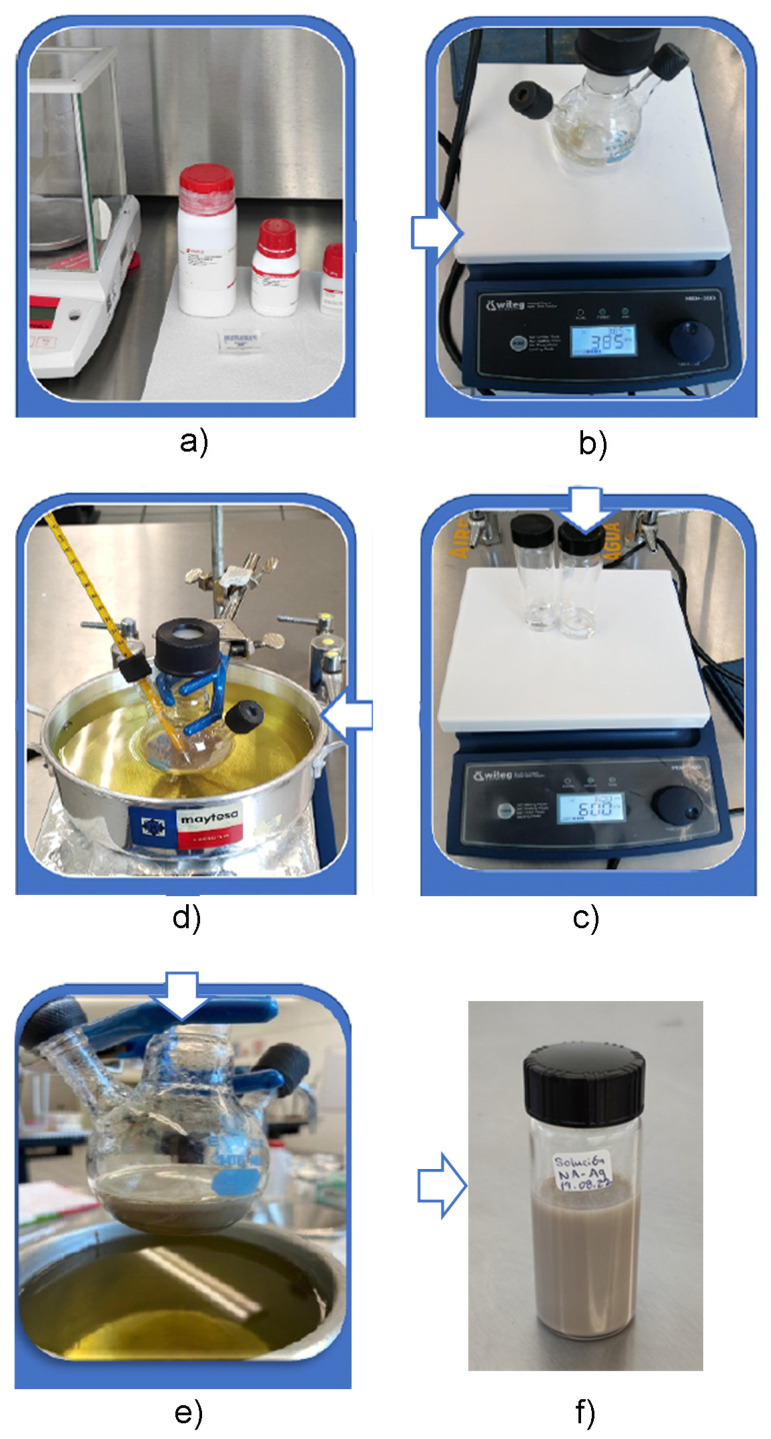
Experimental setup for synthesis of AgNWs. (**a**) Reagents: AgNO_3_, EG, PVP; (**b**) Mixing the solution: PVP-EG; (**c**) Mixing the solutions: NaCl-EG, AgNO_3_-EG; (**d**) Mixing the solutions at 170 °C: PVP-EG, NaCl-EG, AgNO_3_-EG; (**e**) AgNWs solution obtained; (**f**) AgNWs cleaned.

The synthesis of AgNWs was carried out using the polyol chemical method. The experimental setup is presented in Figure 1. The reagents employed in this process included silver nitrate (AgNO_3_), ethylene glycol (EG), polyvinylpyrrolidone (PVP), and sodium chloride (NaCl) [27]. In this synthesis, ethylene glycol acted as the reducing agent for silver ions, while PVP was used as a stabilizer agent for the AgNWs. 

The process was performed by mixing 100 mg of PVP with 15 mL of EG at 600 rpm for 15 min. Also, in separated vials, it was prepared 0.4 g of NaCl with 1 mL of EG, and 90 mg of AgNO_3_ with 5 mL of EG. Both solutions were mixed at 600 rpm for 15 min. Then, the PVP-EG solution was heated at 170 °C in an oil bath on a magnetic stirrer. At this temperature, NaCl-EG and AgNO_3_-EG solutions were mixed constantly in the PVP-EG solution for 1 h. Subsequently, the obtained nanowires underwent washes with acetone (1:4) and ethanol (1:2) to remove residues of ethylene glycol, excess PVP, and possible nanoparticles present in the solution. Finally, a wash with deionized water was carried out to eliminate any residue of sodium chloride (NaCl). 

The synthesized AgNWs were morphologically characterized using a scanning electron microscope (Model JSM-5300, JEOL USA Inc., Peabody, MA, USA). Optical characterization was performed using a UV-Vis spectrophotometer (Model 3101PC, Shimadzu Europe, Manchester, UK).

**Figure 2:**
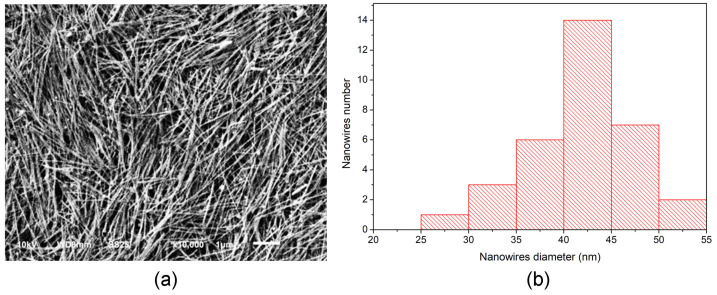
(**a**) SEM image of the AgNWs. (**b**) Size distribution histogram with *X_c_* = 40 nm and standard deviation, σ = 3.4 nm.

Figure 2a presents the SEM image of the AgNWs, showing a high density of nanostructures. In Figure 2b, a histogram of the diameter distribution of the NWs is presented. It is observed that the average diameter (*X_c_*) of the AgNWs is 40 nm, with a standard deviation of σ = 3.4 nm, and lengths reaching the micrometer scale.

**Figure 3:**
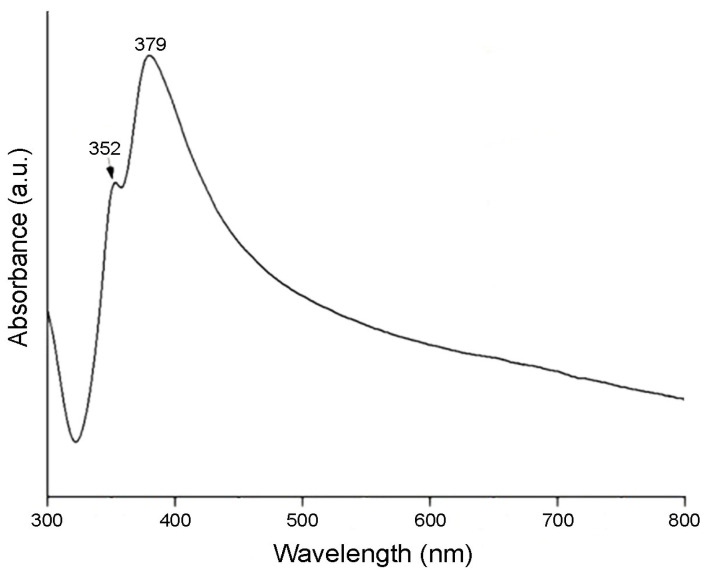
Absorption spectrum of the AgNWs.

Figure 3 shows the absorbance spectrum of the AgNWs versus wavelength after the washing process, showing the characteristic peaks of localized surface plasmon resonance in both transverse (352 nm) and longitudinal (379 nm) modes.

### 2.2. Film Fabrication

**Figure 4:**
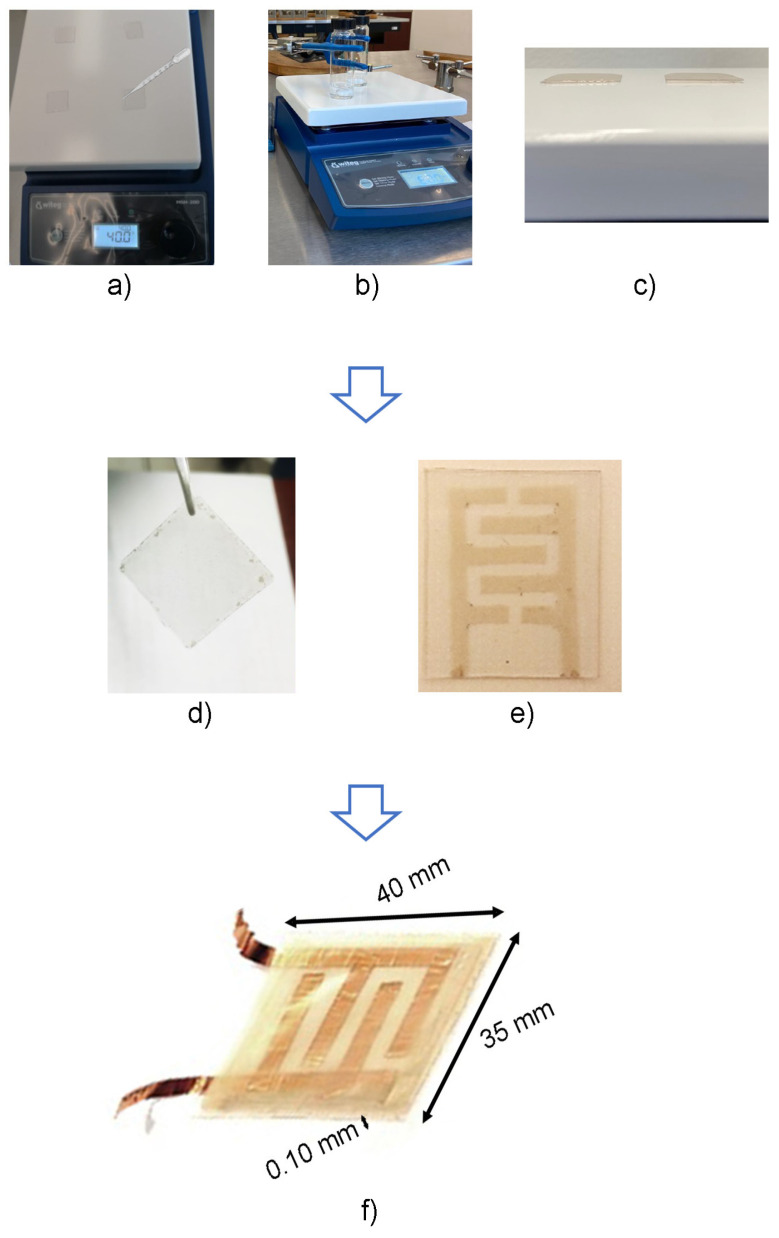
Experimental setup of films fabrication. (**a**) AgNWs dropped on PET substrate at 65 °C for 10 min; (**b**) PVA in 10 mL water at 65 °C for 2 h; (**c**) PVA dropped at PET substrated/AgNWs at 30 °C for 8 h; (**d**) Resistive film; (**e**) Active film with a pattern of AgNWs; (**f**) Fabricated sensor.

The fabrication of flexible nanocomposite films, both resistive and active, was carried out using the drop-casting method. The experimental setup is shown in Figure 4. In this process, polyethylene terephthalate (PET) was used as the substrate, while PVA served as the polymeric matrix for the nanocomposite films. The consideration of using PVA was based on its characteristics as a thermoplastic, reusable, non-toxic polymer, and hydrophilic. Moreover, it has very good mechanical properties and good stability over long periods of time under different temperature and pH conditions. Additionally, PVA is known for being water-soluble, simplifying the film production process, as it does not require additional solvents. For the PVA film fabrication, 0.5 g of PVA was weighed, dissolved in 10 mL of water, and subjected to agitation for 2 h at 60 °C.

**Figure 5:**
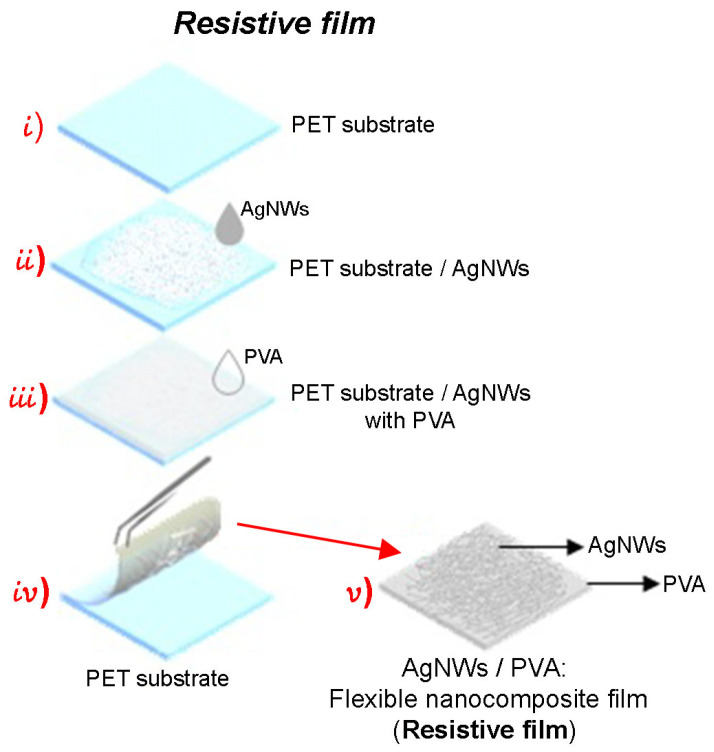
Fabrication process of the resistive flexible nanocomposite film.

The fabrication process of the flexible nanocomposite film (resistive) begins with the deposition of 180 µL of AgNWs solution onto a PET substrate, followed by a drying process by exposing the material to a temperature of 65 °C for 10 min. Subsequently, 360 µL of PVA (0.05 g/mL) is incorporated and allowed to dry at 30 °C for a period of 8 h. Finally, the PVA film with the adhered AgNWs is peeled off from the PET substrate, resulting in the formation of the resistive nanocomposite film: PVA/AgNWs. This process is shown schematically in Figure 5.

**Figure 6:**
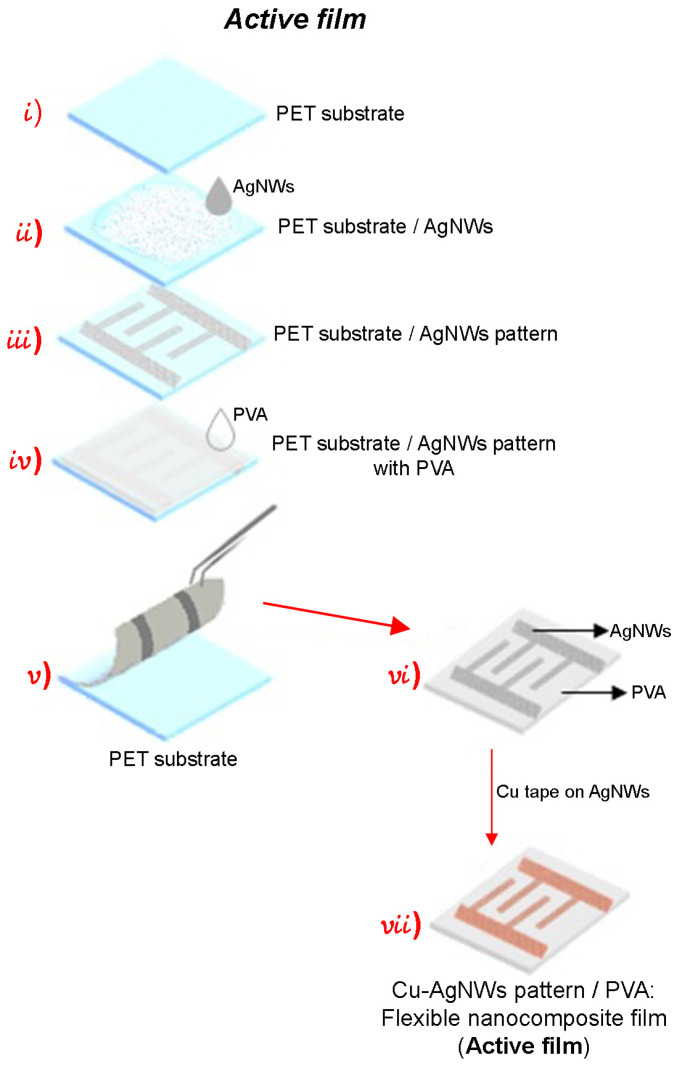
Fabrication process of the active flexible nanocomposite film (electrode).

The fabrication process of the active nanocomposite film or electrode follows a similar procedure to the one described above, as shown in Figure 6. However, in this case, instead of having a film with AgNWs, a pattern was created (see Figure 6 (iii)) with the aim to not sacrifice the flexibility of the film and cover a specific area. Over the AgNWs pattern, it was necessary to employ a copper (Cu) tape to enhance the stability of the electrode response, since the electrode response without the Cu tape was unstable. This instability could be due to the bending effect of the AgNWs on the motion of the electrons and to the discontinuities resulting from the random spatial distribution of the AgNWs, which may lead to a certain rugosity of the film and a consequent decrease in the electron flux. Placing the Cu tape over the AgNWs pattern allows the transfer of electrons towards the tape, which acts as a continuous film, and this makes the motion of the electrons easier in comparison to their motion in the nanowire net itself. Furthermore, establishing an ohmic contact between Ag and Cu favors a good electronic flux between these two materials.

### 2.3. Fabrication of the Force Sensor

**Figure 7:**
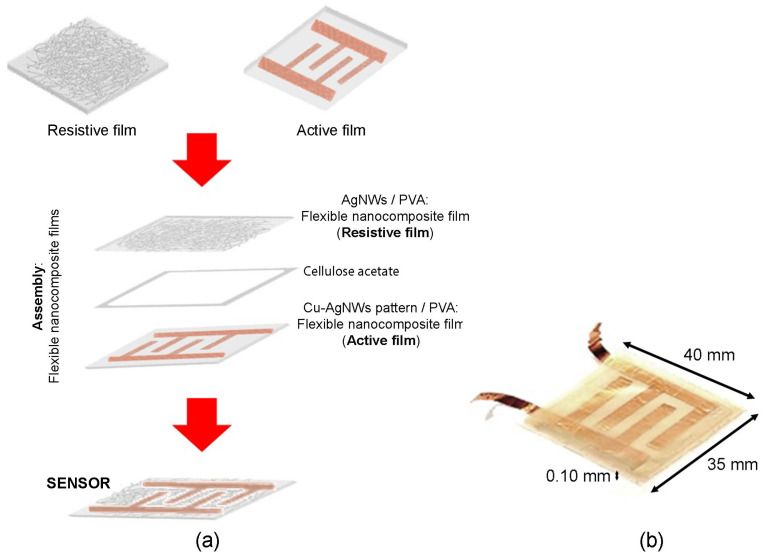
(**a**) Assembly of the resistive and active nanocomposite films of the sensor; (**b**) fabricated real sensor.

Once the flexible nanocomposite films are fabricated, they are carefully assembled. The active and passive films are aligned and placed facing each other. To isolate the two film layers, a cellulose acetate separator frame is inserted between them. This process is illustrated in Figure 7. The result is a force sensor with 35 mm in width, 40 mm in length, and a thickness of 0.10 mm. Figure 7b displays the actual fabricated sensor. The electrical characterization of the sensor was carried out by measuring the voltage change as a function of the applied force or pressure. The sensor response was measured using a digital multimeter and a Wheatstone bridge electronic interface topology.

The calculation of the sheet or frame resistance (*R_s_*) was carried out using the two-point method [28,29]. The choice of this method was based on the homogeneity of the film and the non-uniformity in the distribution of the AgNWs, since these are dispersed randomly, generating areas with a greater or lesser amount of nanowires. This two-point method consists of measuring the resistance (*R*) at the edges or ends of the film, averaging the resistance obtained, and multiplying it by the ratio of the width (W) and length (L) of the film. This is expressed as *R_s_* = *R* (*W*/*L*) [28,29]. In this case, *W* = 35 mm, *L* = 40 mm, and the average resistance measured was 117 Ω, obtaining a value of *R_s_* = 102.4 Ω.

The amount of AgNWs used was 180 µL, as lower quantities did not register an adequate electrical response. Currently, we are not exploring higher loads due to the necessity of maintaining film transparency. An increase in the amount of AgNWs would increase the conductivity of the film but reduce its transparency. Additionally, excessive amounts of AgNWs and PVA could compromise the flexibility of the sensor, making it more rigid.

### 2.4. Sensing and Working Mechanisms

The detection mechanism of the force sensor relies on the change in resistance when the device is subjected to an applied force, a common characteristic of a piezoresistive sensor. This type of device converts the applied force into resistance changes that can be measured or adjusted.

**Figure 8:**
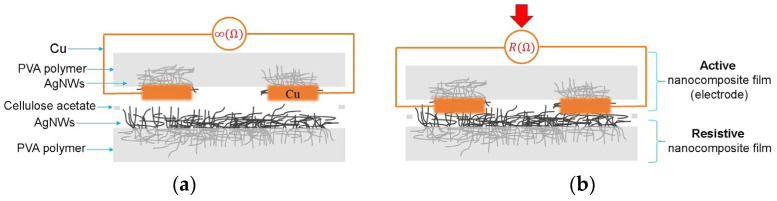
Cross-sectional diagram of the force sensor: (**a**) without pressure and (**b**) with a force applied.

Figure 8a shows the cross-sectional view of the sensor without any applied force, showing infinite resistance. Upon applying force (as depicted in Figure 8b), the nanocomposite films come into contact, inducing a change in the sensor resistance. As the force applied to the sensor increases, the resistance value decreases due to increased contact between the AgNWs. In other words, the minimum resistance value implies maximum contact between the AgNWs. When the force exerted on the sensor is removed, the resistance returns to infinity, and the sensor stops measuring. Thus, the sensor without any applied force remains in an inactive state, resulting in energy savings. Consequently, when the sensor is connected to an electronic interface, it does not consume power.

The electrical properties of the films comprising the sensor are inherently temperature-sensitive, as is common with such materials. However, it is important to note that the temperature coefficient of resistance (TCR) of silver (Ag) and copper (Cu) is in the range of milliohms per degree Celsius (mΩ/°C), specifically 3.8 mΩ/°C and 4.3 mΩ/°C, respectively [30]. Thus, the sensor is expected to maintain its functionality and performance within a reasonable range of ambient temperature variations. Regarding the PVA, the TCR for this material is lower than that of AgNWs.

In a bridge signal conditioner, typically excitation voltage levels of around 3 V and 10 V are common. A higher output voltage can cause larger errors because of self-heating. Otherwise, an unstable excitation source can affect the accuracy of measurements [31]. 

If a high level of excitation were applied, the developed sensor would experience heating in the conductors and connection strips. This phenomenon would affect the system by changing the resistivity and sensitivity properties of the bridge components, as well as the polymer structure of the sensor or measurement device. Consequently, this adverse effect would result in a degradation of sensor performance. 

Initially, the bridge was calibrated to achieve optimal sensor performance. In this procedure, all three potentiometers of the bridge were adjusted to 0 Ω. Subsequently, the circuit was connected to the power supply, and the bridge output terminals were connected to a multimeter to monitor variations. A minimum force of 0.039 N was applied to the sensor, following which the value of each potentiometer was increased, and the voltage variation at the sensor output was measured until it did not exceed 2 V. The applied force was gradually increased, ensuring that the voltage obtained with the minimum force did not reach its maximum value, which, in this case, was 5 V. Multiple calibrations were performed until the bridge values with the best performance were achieved.

**Figure 9:**
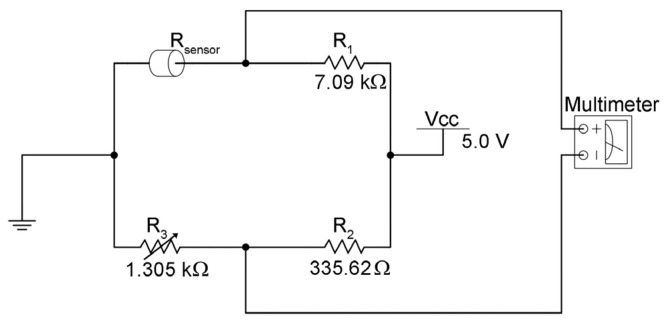
Signal conditioning circuit using Wheatstone bridge topology. R_1_, R_2_ and, R_3_ are the precision potentiometers, with R_3_ as a variable resistance; R_sensor_ is the resistance generated by the sensor; V_cc_ is the voltage applied. The electrical response is measured using a multimeter.

The electrical response characterization of the sensor was performed through voltage measurements as a function of the applied force. The sensor terminals were connected to a Wheatstone bridge electronic interface to adjust resistance values, as shown in Figure 9.

The Wheatstone bridge was implemented using precision potentiometers in a range from 2 Ω to 100 kΩ, allowing for sensor calibration based on the required output voltage values for measurement readings, with an excitation level of 5 V. 

## 3. Results and Discussions

**Figure 10:**
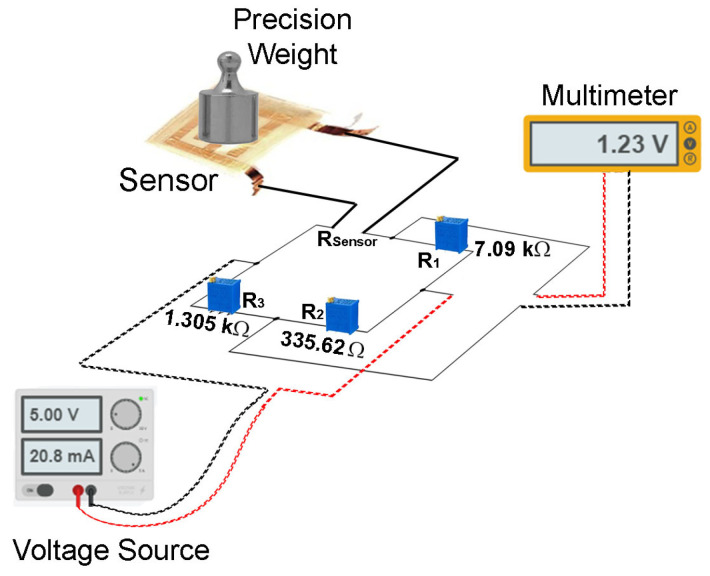
Experimental setup scheme for sensor characterization.

The sensor was characterized using the experimental setup shown in Figure 10. The sensor terminals were connected to the Wheatstone bridge, to which a constant voltage of 5 V was applied through a voltage source (Model 1670A, BK PRECISION, Yorba Linda, CA, USA). The electrical response measurements were carried out using a digital multimeter (Model 115, FLUKE, Everett, WA, USA), with which the voltage change was recorded as a function of the applied force. The force application on the sensor was carried out using precision weights placed on the device. The results indicated that a minimum force of 0.039 N (equivalent to 4 g) was required to obtain a sensor response. Then, a gradual increase in the applied weight was performed until completing the full operational range of the sensor.

**Figure 11:**
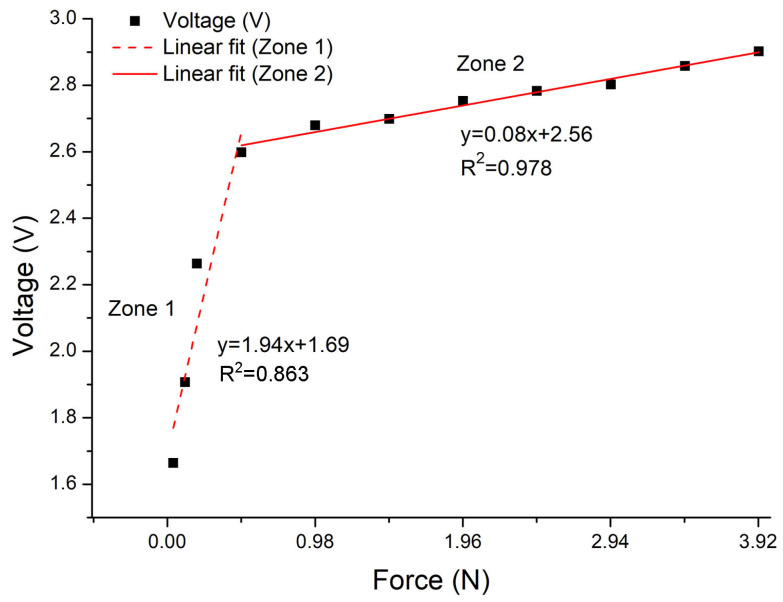
Voltage behavior as a function of the applied force, and linear adjustment in the measuring range of the force sensor.

Figure 11 shows the results of the electrical characterization, demonstrating the voltage change recorded as a function of the applied force and the typical behavior of a piezoresistive sensor. The obtained results show that in the absence of an applied force, the voltage is zero because the sensor exhibits infinite resistance. Upon exerting force on the sensor, different voltage values are recorded in response.

The first voltage response is obtained with an applied force of 0.039 N (R = 50 MΩ), generating a voltage response of 1.66 V. As the applied force increases, the voltage values also increase. The maximum measured voltage value, 2.9 V, is reached with an applied force of 3.92 N, considered as the sensor saturation point. It was confirmed that for an applied force of 4.41 N, the same voltage value is obtained as for 3.92 N. The voltage decreases for a force of 4.90 N. Therefore, the effective operating range of the sensor is considered from 0.039 N to 3.92 N.

Figure 11 also shows the linear fit of the voltage response as a function of the applied force within the sensor operating range. Two linear zones are observed: the first zone (Zone 1) with a slope of 1.94 and a second one (Zone 2) with a slope of 0.08. In this graphic, it is demonstrated that the correlation coefficient (R^2^) obtained in Zone 2 is 0.976 and in Zone 1 is 0.863. The value for Zone 2 is closer to one, which indicates that there is a better fit in this zone than in Zone 1.

The behavior of Zone 1 can be attributed to the limited contact between the two films. As the applied force increases, the films have better contact with each other, resulting in a better linear response (Zone 2). It is considered that the behavior of the response within the sensor operating range is due to both the heterogeneousness and randomness of the distribution of AgNWs in the film. The aforementioned would mean a certain roughness or porosity in the film.

The electrical characterization allowed determining the values of the most significant figures of merit of the piezoresistive sensor at room temperature, such as the actuation force, stand-off resistance, working range, response time, stabilization time, sensitivity, hysteresis degree, and sensor repeatability.

The actuation force, defined as the minimum force that causes a change in the sensor output, was measured at 0.039 N, which is the value that triggers a response in the sensor. The stand-off resistance exceeded 50 MΩ, which represents the sensor resistance when a force is not applied on it. The operating range, defined by the minimum and maximum values of the physical variable that the sensor can measure, was determined from 0.039 N to 3.92 N. The sensor response time was 10 ms, indicating the minimum time to record a change in output after applying a force. Then, the sensor bandwidth is 100 Hz. The stabilization time is known as the period during which the sensor stabilizes after receiving a force. This figure of merit was recorded at 9 s, during which output variations were minimal. The sensitivity of the sensor, defined as the minimum input value required to produce a change in the output, was set at 0.039 N.

Additionally, the degree of hysteresis (*DH*) of the sensor was calculated using the following equation:

(1) $$DH=\frac{v_{2}-v_{1}}{v_{max}-v_{min}}\times 100\left(\%\right),$$

where *v_2_* and *v*_1_ are the values of the voltage obtained for a given value of the force, when the force applied to the sensor increases (loading) from a minimum to a maximum value and then decreases (unloading) from the maximum to the minimum value. In this equation, *v_min_* and *v_max_* are the output voltages for the minimum and maximum forces, respectively.

**Figure 12:**
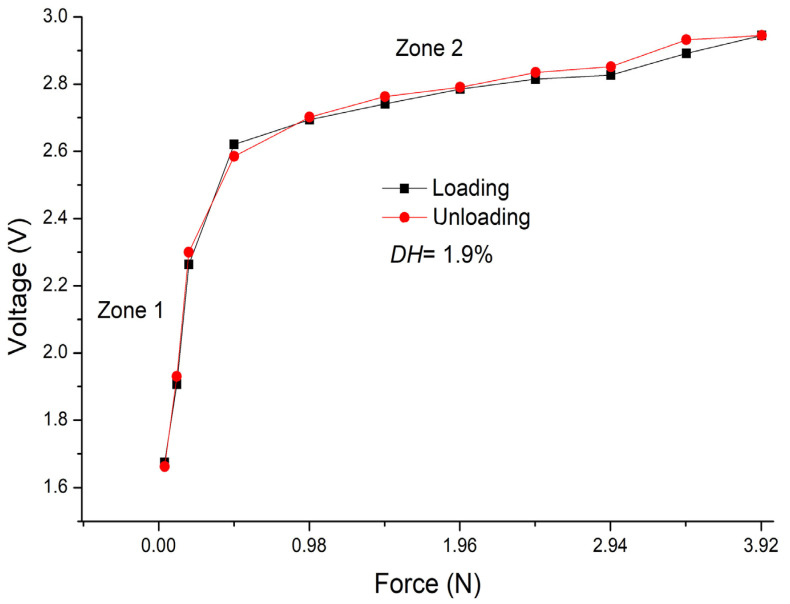
Sensor hysteresis curve.

The calculation of the sensor hysteresis degree was *DH* = 1.9%, obtained by averaging each measured force value (Figure 12). This is an important parameter, since a lower value of *DH* indicates smaller variations in the measurement parameters, independently of the measurement direction (loading or unloading), and indicates a smaller effect of lag or memory in the device. With the aim to validate the hysteresis value, twenty loading/unloading tests were carried out. This highlights the remarkable viscoelasticity of the sensor materials, which exhibit an elastic response when deformed under the application of force. 

**Figure 13:**
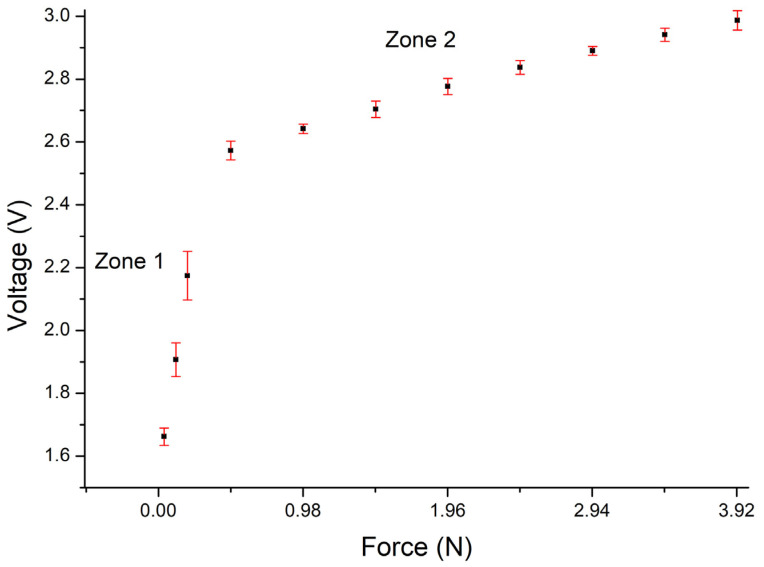
Sensor repeatability measurement error graph.

Figure 13 shows the repeatability error across the entire sensor operating range (Zone 1 and Zone 2), derived from 20 measurements. It can be observed that the repeatability error in Zone 1 is higher than in Zone 2, which is attributed to the better linear response in Zone 2, as indicated by its correlation constants. Similarly, in the last two measurements (higher force), although the error is not significant, there is a slight overlap of the measured values, which is due to reaching the sensor maximum measurement range.

**Figure 14:**
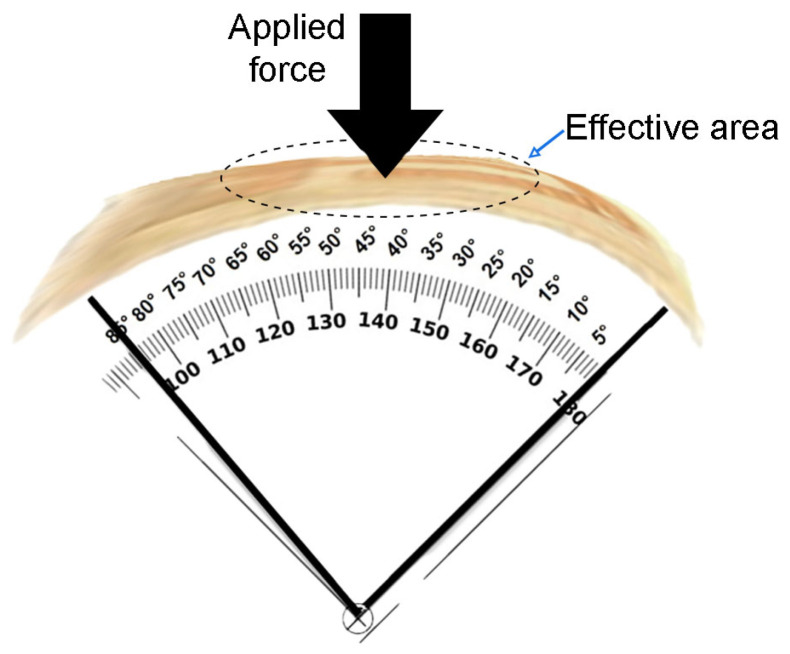
Scheme of the sensor bending at an angle of 85°. The dots line indicates the reduction of the measurement effective area on the sensor under bending.

Flexible devices, such as those reported in this work, must have the capability to withstand bending and remain functional. Therefore, the flexibility of the sensor was verified by applying a bending force at an angle of 85° for 60 min. The sensor was fixed over a cylindrical surface. The scheme of the sensor bending at an angle of 85° is shown in Figure 14. It is observed the reduction in the effective area due to the bending where the force can be applied.

**Figure 15:**
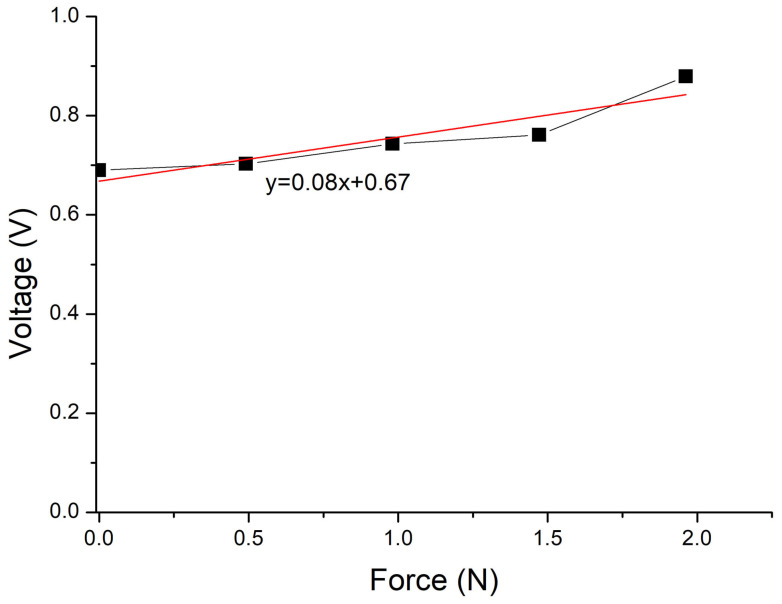
Voltage response as a function of the applied force on the sensor bending at an angle of 85°. The red line is the linear fit.

Figure 15 shows the results of the voltage behavior as a function of the applied force on the effective area on the sensor bending at an angle of 85°. It is observed that without the application of force, the sensor responds with a voltage value of 0.690 V. This is because the resistive and active films of the sensor are in contact due to the bending. For sensor applications, this measured voltage value under bending could be considered its offset.

When a variable force is applied to the sensor from 0.5 N, an increase in voltage can be observed. As the applied force increases, the voltage slightly rises at a rate of 0.08 V/N. Note that despite the flexible state of the sensor, the stability or slope of 0.08 V/N is maintained. However, it is noticeable in the graph that the number of measured points is lower, as after the force of 2.0 N, the voltage drops, reaching sensor saturation. Thus, the operating range of the sensor under flexion is reduced. This behavior could be attributed to the effective area of the sensor being reduced (Figure 14), meaning that the measurement area where the force is applied on the sensor is smaller due to the sensor being flexed. Figure 15 shows a linear fit of the voltage response as a function of the force of the sensor under flexion. The linearity of the measured points is demonstrated. 

It is worth mentioning that the sensor returns to its original shape after being bent to confirm its functionality and response. It was obtained the same response as in Figure 11, measuring an infinite resistance without a force applied on the sensor, and obtaining the same voltage values when the force increases.

One of the important ideas in the study and manufacturing of this sensor prototype is the possible applications it can have. However, a particular interest lies in sensing small force ranges, aiming to apply them in systems with limited and confined spaces, such as measuring forces in knee or elbow joints in robot movements. Additionally, the angle of bending applied to the sensor (at 85°) was considered, as it is a relevant angle in the study and development of knee movements, as well as evaluating the voltage response in relation to the sensor force over a certain period when it is under bending.

The ability to measure small forces in knee or elbow joints is crucial for various applications in robotics and biomechanics. In robotic systems, accurate force measurements are essential for tasks requiring delicate movements and interactions with the environment. For example, in prosthetic limbs or exoskeletons, precise force-sensing enables natural and responsive movements, enhancing user comfort and safety [32]. Additionally, in rehabilitation robotics, measuring forces exerted on joints during physical therapy sessions allows for personalized treatment plans and progress tracking [33,34]. Therefore, by incorporating sensors capable of measuring small forces, robotic systems can achieve greater precision and adaptability in performing tasks involving joint movements.

In comparison with force or pressure sensors reported and based on materials similar to those used in this work, such as silver (Ag) and polymers, the response time that is present is longer (20 to 50 ms) than that obtained with the developed sensor [35,36,37]. Moreover, although sensors have been reported that use the same operating mechanism with a wider range of measured forces or lower response time, the manufacturing techniques of these sensors are usually more complex and expensive [38,39].

With the aim of improving the sensor linear response, it will be necessary to implement compensation algorithms that can be used in conjunction with the development of user-friendly interfaces that facilitate feedback and data management from the sensor. Additionally, in order to expand the potential application of the sensor, a review of the design will be required to assess the possibility of extending the range of measured forces.

## 4. Conclusions

In this work, a prototype force sensor was fabricated using flexible nanocomposite films (PVA/AgNWs) through a simple and cost-effective method. The nanocomposite films were fabricated using a thermoplastic (PVA) because it is a reusable, non-toxic, hydrophilic polymer with good mechanical properties and is stable for long periods under different temperature and pH conditions. AgNWs with diameters of 42 nm were used as the conductive filler material in both the resistive and active (electrode) films. The fabricated sensor measured 35 mm in width, 40 mm in length, and had a thickness of 0.10 mm. The characterization of the sensor demonstrated a sensitivity ranging from 0.039 N to 3.92 N, with a stand-off resistance greater than 50 MΩ, response time of 10 ms, stabilization time of 9 s, hysteresis value of 1.9%, and a very good repeatability. The bending test of the sensor, with an angle to 85° of bending for 60 min, verified that the sensor maintained its properties by preserving its measuring functionality. This suggests the possible extension of the use of the device for pressure measurements when placed over other not-so-regular surfaces. 

The fabricated sensor can be used in applications that require measuring pressure on irregular surfaces or systems with limited space, such as in estimating movement in robotics and biomechanics. Regarding the nanocomposites applied in this work, these have potential applications as flexible, transparent, conductive electrodes. Due to its optimal electrical and optical properties, this material can be used in a variety of areas unrelated to sensors, such as solar cells, smart windows, and smart screens.

## Data Availability

The raw data supporting the conclusions of this article will be made available by the authors on request.
